# Dissociated responses at initial computed tomography evaluation is a good prognostic factor in non-small cell lung cancer patients treated with anti-programmed cell death-1/ligand 1 inhibitors

**DOI:** 10.1186/s12885-020-6704-z

**Published:** 2020-03-12

**Authors:** Takehiro Tozuka, Satoru Kitazono, Hiroaki Sakamoto, Hiroshi Yoshida, Yoshiaki Amino, Shinya Uematsu, Takahiro Yoshizawa, Tsukasa Hasegawa, Ken Uchibori, Noriko Yanagitani, Atsushi Horiike, Takeshi Horai, Masahiro Seike, Akihiko Gemma, Makoto Nishio

**Affiliations:** 1 Department of Thoracic Medical Oncology, The Cancer Institute Hospital, Japanese Foundation for Cancer Research, Tokyo, Japan; 2 Department of Pulmonary Medicine and Oncology, Graduate School of Medicine, Nippon Medical School, Tokyo, Japan

**Keywords:** Non-small cell lung cancer, Immune checkpoint inhibitors, Dissociated responses

## Abstract

**Background:**

Dissociated responses (DR) are phenomena in which some tumors shrink, whereas others progress during treatment of patients with cancer. The purpose of the present study was to evaluate the frequency and prognosis of DR in non-small cell lung cancer (NSCLC) patients treated with anti-programmed cell death-1/ligand 1 (anti-PD-1/L1) inhibitors.

**Methods:**

This retrospective study included NSCLC patients who received anti-PD-1/L1 inhibitor as second- or later-line treatment. We excluded patients without radiological evaluation.

In patients who showed progressive disease (PD) according to the RECIST 1.1 at the initial CT evaluation, we evaluated all measurable lesions in each organ to identify DR independently of RECIST 1.1. We defined DR as a disease with some shrinking lesions as well as growing or emerging new lesions. Cases not classified as DR were defined as ‘true PD’. Overall survival was compared between patients with DR and those with true PD using Cox proportional hazards models.

**Results:**

The present study included 62 NSCLC patients aged 27–82 years (median: 65 years). DR and true PD were observed in 11 and 51 patients, respectively. The frequency of DR in NSCLC patients who showed PD to anti-PD-1/L1 was 17.7%. Median overall survival was significantly longer in patients with DR versus true PD (14.0 vs. 6.6 months, respectively; hazard ratio for death: 0.40; 95% confidence interval: 0.17–0.94).

**Conclusions:**

Patients with DR exhibited a relatively favorable prognosis.

## Background

Treatment with anti-programmed cell death-1/ligand 1 (anti-PD-1/L1) inhibitors has demonstrated survival benefit in patients with non-small cell lung cancer (NSCLC). The administration of nivolumab, pembrolizumab, and atezolizumab resulted in improved overall survival (OS) in patients with advanced NSCLC after failure of first-line treatment [1–4]. The response patterns of tumors in patients treated with anti-PD-1/L1 inhibitors may differ from those observed in patients treated with conventional cytotoxic agents owing to their characteristic mechanism.

Atypical patterns of response to anti-PD-1/L1 inhibitors have been reported, including durable responses, pseudoprogression, hyperprogression, and dissociated responses (DR). Durable responses are continuous responses to anti-PD-1/L1 inhibitors even after discontinuation of treatment [5]. Pseudoprogression is observed as objective response following temporary tumor growth [6]. Hyperprogression refers to rapid disease progression after the initiation of immunotherapy [7]. DR are phenomena in which several tumors shrink, whereas others progress [8].

DR are also considered mixed responses in NSCLC patients. A previous study reported that the overall incidence of mixed responses or DR in NSCLC patients treated with systemic therapy was 21.5% [9]. Tumor heterogeneity within individual patients may be responsible for these inconsistent responses to treatment. From another point of view, DR may be explained based on differences in tissue penetration by drugs in each organ. Notably, DR has been reported to be an unfavorable prognostic factor of survival [9].

However, the frequency of DR at the initial computed tomography (CT) evaluation is unclear in NSCLC patients receiving treatment with anti-PD-1/L1 inhibitors. In addition, the prognosis of patients who experienced DR to anti-PD-1/L1 inhibitors remains undetermined. The aim of the present study was to identify radiological features of NSCLC patients who achieved survival benefit of anti-PD-1/L1 inhibitors and showed PD to this type of therapy.

## Methods

### Patients

We retrospectively reviewed the clinical data of consecutive patients with histologically confirmed advanced NSCLC who received anti-PD-1/L1 inhibitor monotherapy (nivolumab, pembrolizumab, or atezolizumab) as second- or later-line treatment at the Cancer Institute Hospital, Japanese Foundation for Cancer Research, (Tokyo, Japan) between December 2015 and December 2018. All patients received nivolumab (3 mg/kg of body weight or 240 mg per body) every 2 weeks, pembrolizumab (200 mg per body) every 3 weeks, or atezolizumab (1200 mg per body) every 3 weeks. We excluded patients who were not evaluated using chest CT, abdominal CT, and brain imaging (CT or magnetic resonance imaging) within 28 days prior to the initiation of immunotherapy. We also excluded patients without measurable lesions and those who did not undergo CT evaluation after the initiation of treatment with anti-PD-1/L1 inhibitors. We analyzed the patients whose initial response to anti-PD-1/L1 inhibitors was assessed as progressive disease (PD) according to the Response Evaluation Criteria in Solid Tumors version 1.1 (RECIST 1.1) at the initial CT evaluation [10]. The study protocol was reviewed and approved by the Ethics Committee of the Cancer Institute Hospital, Japanese Foundation for Cancer Research (approval number 2019–1073).

### Evaluation of efficacy

Objective response at the initial CT evaluation was assessed as complete response, partial response (PR), stable disease (SD), or PD in accordance with RECIST 1.1 [10]. In patients who showed progressive disease (PD) according to the RECIST 1.1 at the initial CT evaluation, we evaluated all measurable lesions in each organ to identify DR independently of RECIST 1.1. We defined DR as a disease with some shrinking lesions as well as growing or emerging new lesions. PD not classified as DR was defined as true PD.

OS was defined as the time from the initiation of anti-PD-1/L1 inhibitor monotherapy to the date of death. OS was compared between patients who exhibited DR and those who presented true PD. We defined time to treatment failure as the time from the first dose of anti-PD-1/L1 inhibitors to the discontinuation of treatment for any reason, including disease progression, treatment toxicity, patient request, or death. The duration of treatment beyond progression was calculated as the time from the evaluation of disease progression to discontinuation of treatment for any reason.

### Statistical analysis

We used Fisher’s exact test to compare patient characteristics between different groups. OS was compared using the Kaplan–Meier method. The hazard ratio was calculated using Cox proportional hazards models. A *p* <  0.05 denoted statistical significance. All significant factors identified in the univariate analysis were entered in the multivariate analysis. All statistical analyses were performed using the EZR® version 1.37 software (Saitama Medical Center, Jichi Medical University, Saitama, Japan), which is a graphical user interface for R (The R Foundation for Statistical Computing, Vienna, Austria) [11].

## Results

### Patients

**Fig. 1:**
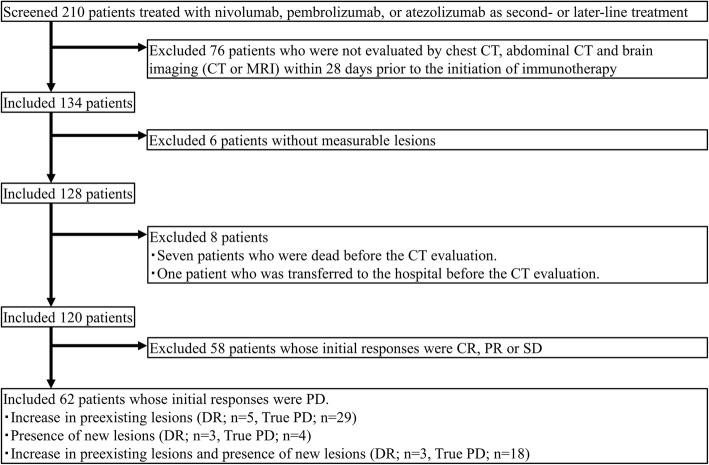
Patient selection flow. Abbreviations: CR; complete response; PR, partial response; SD, stable disease; DR, dissociated responses; true PD, true progressive disease

A total of 210 patients received anti-PD-1/L1 inhibitor monotherapy (nivolumab, pembrolizumab, or atezolizumab) as second- or later-line treatment. Of those, 120 patients who were radiologically evaluable, and 62 patients assessed as PD at the initial CT evaluation according to the RECIST 1.1 were enrolled in this analysis (Fig. 1). Patient characteristics are shown in Table 1. The median age was 65 years (range: 27–82 years). Most patients were aged < 75 years (*n* = 51) and 40 patients were male. The majority of patients had adenocarcinoma (*n* = 46). Of note, 15 patients were positive for driver mutations, including 12 patients with epidermal growth factor receptor mutation and three patients with anaplastic lymphoma kinase rearrangement. PD-L1 expression was evaluated in 36 patients. Among all patients, 45, 7, and 10 patients received nivolumab, pembrolizumab, and atezolizumab, respectively.

**Table 1:** Patient characteristics

		All (*n* = 62)	DR (*n* = 11)		True PD (*n* = 51)		*P*
		n	n	%	n	%	
Age	median 65, range 27–82						
	≥75	11	2	18	9	18	1.000
	< 75	51					
Gender	Male	40	7	64	33	65	1.000
	Female	22					
Performance status	0,1	46	8	73	38	75	1.000
	2–4	16					
Smoking status	Current/former	45	6	55	39	76	0.155
	Never	17					
Histology	Squamous cell carcinoma	12	2	18	10	20	1.000
	Non-squamous cell carcinoma	50					
	Adenocarcinoma (*n* = 46), Others (*n* = 4)						
Driver mutation	Positive	15	3	27	12	24	0.696
	EGFR L858R/19del (*n* = 12), ALK (*n* = 3)						
	Negative	47					
Line of treatment	2nd line	32	7	64	25	49	0.511
	3rd line or beyond	30					
PD-L1 status	< 1	8	0	0	8	16	
	1 ≤	28	7	64	21	41	
	Unknown	26	4	40	22	43	
Treatment	Nivolumab	45	9	20	36	80	
	Pembrolizumab	7	2	29	5	71	
	Atezolizumab	10	0	0	10	100	

*Abbreviations*: *DR* dissociated responses, *PD-L1* programmed cell death-ligand 1, *True PD* true progressive disease

### Dissociated responses

**Fig. 2:**
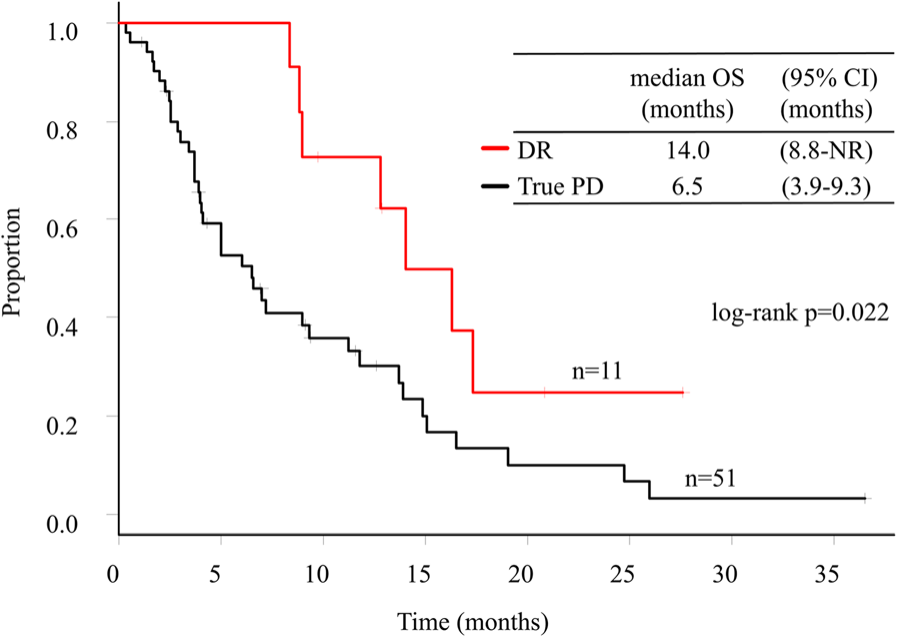
Overall survival in patients with dissociated responses and true progressive disease. Abbreviations: DR, dissociated responses; True PD, true progressive disease

**Fig. 3:**
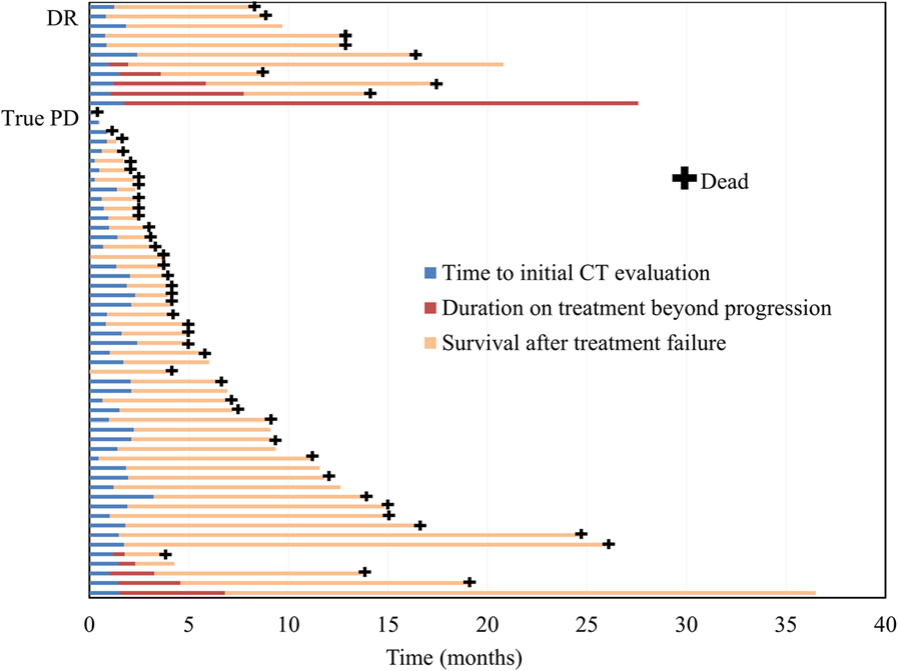
Swimmer plots showing time to initial CT evaluation, duration of treatment beyond progression, and survival time after treatment failure. Abbreviations: DR, dissociated responses; True PD, true progressive disease

The interval between the first dose of anti-PD-1/L1 inhibitors and initial CT evaluation was usually ≤2 months. Based on the evaluation of all lesions at the initial CT, of the 62 patients assessed as PD according to the RECIST 1.1, 11 patients (17.7%) exhibited DR. Among those, nine patients were treated with nivolumab and two patients were treated with pembrolizumab. Median OS was significantly longer in patients assessed as DR than in those assessed as true PD (14.0 vs. 6.5 months, respectively; hazard ratio for death: 0.40; 95% confidence interval: 0.17–0.94) (Fig. 2). Figure 3 shows the time course from the initial dose of anti-PD-1/PD-L1 inhibitors until death for each patient.

Among the 62 patients who were assessed as PD, five of the 11 patients (45.4%) with DR and five of the 51 patients (9.8%) with true PD continued treatment with anti-PD-1/L1 inhibitors. Three patients were treated with other anticancer agents, and one patient received local radiotherapy. One patient received local radiotherapy and another anticancer agent, while another patient received best supportive care.

The median interval between the initial dose of anti-PD-1/L1 inhibitors and initial CT evaluation was 44 days (range: 4–72 days). Median time to treatment failure of anti-PD-1/L1 inhibitors in patients with DR and true PD was 5.9 and 3.3 months, respectively. Median duration of treatment beyond progression in five patients with DR and five patients with true PD was 4.6 months and 2.2 months, respectively. Especially, the administration of anti-PD-1/L1 inhibitors was continued for > 6 months beyond progression in three of the five patients (60%) with DR and in only one of the five patients (20%) with true PD.

In the univariate analysis, there were no significant differences observed in characteristics between patients assessed as DR and those assessed as true PD (Table 1). In the multivariate analysis for OS, performance status and DR were significant prognostic factors (Table 2). Details of the response sites and progression sites are shown in Table 3. Five patients exhibited progression of preexisting lesions, three patients experienced emergence of new lesions, and three patients demonstrated both.

**Table 2:** Univariate and multivariate analyses of overall survival

			Univariate analysis			Multivariate analysis		
Variables		n	HR	95% CI	*P*	HR	95% CI	*P*
Age	< 75 vs ≥ 75	51/11	0.70	0.32, 1.55	0.386			
Gender	Male vs Female	40/22	0.70	0.37, 1.26	0.235			
PS	0, 1 vs 2–4	46/16	0.25	0.13, 0.48	< 0.001	0.18	0.09, 0.36	< 0.001
Smoking	Never vs Current/Ex	17/45	1.05	0.55, 1.99	0.889			
Histology	Sq vs non-Sq	10/52	0.93	0.46, 1.89	0.846			
Driver mutation	Negative vs Positive	47/15	0.59	0.28, 1.23	0.162			
Treatment line	2nd vs 3rd line	32/30	1.11	0.62, 1.99	0.715			
PD-L1^a^	Positive vs Negative	30/8	0.61	0.22, 1.69	0.339			
Response	DR vs True PD	11/51	0.40	0.18, 0.90	0.027	0.28	0.12, 0.64	0.003

Abbreviations: *95% CI* 95% confidence interval, *DR* dissociated responses, *HR* hazard ratio for death, *PD-L1* programmed cell death-ligand 1, *PS* performance status, *Sq*, squamous cell carcinoma, *True PD* True progressive disease

^a^PD-L1 positive; PD-L1 ≥ 1%, PD-L1 negative; PD-L1 < 1%

**Table 3:** Sites of response and progression in patients with dissociated responses, and treatment after dissociated responses in eleven patients with dissociated responses

Age (range)	Sex	ICIs	Treatment after DR	Response site	Progression site
71–75	M	Nivo	Continuation of ICIs	primary lesion	bone; new lesion
26–30	M	Nivo	Continuation of ICIs	primary lesion, LN (thoracic), kidney, adrenal, PM	PM; non-target lesion adrenal; new lesion
46–50	M	Nivo	Continuation of ICIs	PM	primary lesion; target lesion
71–75	M	Pemb	S1 + Bevacizumab	PM	PM; target lesion LN (thoracic);non-target lesion Liver; non-target lesion
66–70	M	Nivo	Continuation of ICIs	primary lesion, LN (thoracic)	adrenal; new lesion
76–80	M	Nivo	BSC	LN (axilla)	PM; new lesion
46–50	F	Nivo	Local radiotherapy	LN (abdominal), LN (axilla)	PM; target lesion LN (thoracic); non-target lesion Subcutaneous metastasis; non-target lesion
51–55	F	Pemb	S1	PM	primary lesion; target lesion PM; new lesion
41–45	F	Nivo	Pemetrexed and local radiotherapy	LN (axilla) PM	Brain; new lesion, PM; new lesion Bone; non-target lesion
76–80	M	Nivo	Continuation of ICIs	primary lesion, PM	LN (thoracic); non-target lesion
66–70	F	Nivo	S1	liver	primary lesion; non-target lesion PM; non-target lesion LN (thoracic); non-target lesion Liver; new lesion, LN (abdominal); new lesion

*Abbreviations*: *BSC* best supportive care, *DR* dissociated responses, *F* female, *ICIs* immune checkpoint inhibitors, *LN* lymph nodes, LN, *M* male, *Nivo* nivolumab, *Pemb*, pembrolizumab, *PM* pulmonary metastasis

## Discussion

Through evaluation of all lesions at the initial CT, DR were identified in 17.7% of NSCLC patients assessed as PD according to the RECIST 1.1. In addition, the survival of patients with DR was significantly longer than that observed in patients with true PD.

The rate of DR in this study was higher than that reported in a previous study (7.5%) investigating NSCLC patients treated with anti PD-1/PD-L1 inhibitors [8]. However, the rate of DR in this study was calculated in patients with PD assessed using the RECIST 1.1. The rate of DR among the 120 NSCLC patients treated with immune checkpoint inhibitors was 9.2%, and this rate was similar to that reported in a previous study (8).

To date, predictors of DR have not been identified in patients treated with anti-PD-1/PD-L1 inhibitors [12]. Although the present study did not identify significantly different factors between patients assessed as DR and those assessed as true PD, performance status and DR were significant prognostic factors of OS in the multivariate analysis. This finding suggested that DR may be a useful marker in determining whether to continue or discontinue the administration of anti-PD-1/L1 inhibitors following the detection of PD by the RECIST during treatment.

The sites of response and progression in patients assessed as DR were not specific. However, the most common sites in which size change was observed were the lymph nodes. The lymph nodes are immunologically privileged sites, and play key roles in the regulation of immune responses to pathogens and autoantigens [13]. Hence, the responses of lymph nodes may differ from those of tumors. Effector T cells and memory T cells are generated after priming of naïve T cells in lymph nodes [14]. While malignant lymph nodes may restrict cytotoxic activity and induce an immunosuppressive environment within them [15], their cytotoxic activity and response remain unknown.

Although the mechanism of DR is unclear, intra-tumoral heterogeneity and differences in the tumor microenvironments between metastatic sites may be responsible. The expression of PD-L1 in tumors is a biomarker for the use of PD-1 inhibitors. Previous studies showed that PD-L1 expression was discordant between tumor specimens obtained from two different sites in 17.1–24.8% of patients [16, 17]. Another study reported that tumor microenvironments influenced the outcome of immunotherapy and differed across organs in patients with cancer [18].

In the present study, the median OS in patients with DR was 14.0 months. This was comparable to the median OS (9.2–17.3 months) of the overall population reported in several clinical trials investigating anti-PD-1/PD-L1 inhibitors [1–4]. In the present study, the survival of patients with DR was significantly longer than that observed in patients with true PD. These results suggested that the survival benefit of immunotherapy may be underestimated by conventional radiological evaluation using RECIST 1.1 [19]. New criteria, such as the immune-related response criteria, immune-related RECIST, immune RECIST, and immune-modified RECIST were proposed to evaluate the response and survival benefit associated with anti-PD-1/L1 inhibitors [19–22]. However, DR are not defined in these new criteria. The median OS in patients with DR in the present study was comparable to that reported in patients with PR or SD in a previous study [23]. Therefore, defining DR in addition to complete response, PR, SD, and PD through radiological evaluation is necessary for the accurate assessment of the efficacy of anti-PD-1/L1 inhibitors.

At present, prompt treatment after DR has not been established. In several clinical trials and current clinical practice, immunotherapy may be continued beyond disease progression (according to the RECIST 1.1), if the attending physicians consider that the treatment offers clinical benefit to the patient [4, 24]. However, the criteria for the continuation of treatment with anti-PD-1/L1 inhibitors beyond progression remain unclear. Although there was no statistically significant difference noted in the duration of treatment beyond progression, three of the five patients with DR and one of the five patients with true PD continued therapy for > 6 months beyond progression. Therefore, DR may be a useful factor in deciding the continuation of treatment with anti-PD-1/L1 inhibitors. However, further studies are warranted to determine the optimal treatment after DR to anti-PD-1/L1 inhibitors.

There were several limitations in the present study. Firstly, this was a retrospective, single-institution study with a small sample size. Additional clinical data are required to identify the optimal radiological evaluation method for determining the clinical benefit of anti-PD-1/L1 inhibitors. Secondly, the optimal interval between the first dose of anti-PD-1/L1 inhibitors and initial CT evaluation remain unknown because they were not standardized in this study. However, in the present study, the median interval between the initiation of treatment with anti-PD-1/L1 inhibitors and initial CT evaluation was 44 days. The interval between pretreatment radiological evaluation (including brain imaging) and the first dose of anti-PD-1/L1 inhibitors in all patients was ≤28 days. These intervals are considered to be reasonable in clinical practice.

## Conclusion

In conclusion, DR to treatment with anti-PD-1/L1 inhibitors was observed in 17.7% of NSCLC patients assessed as PD according to the RECIST 1.1 at the initial CT evaluation. These patients showed relatively favorable prognosis. DR at the initial CT evaluation may be a useful factor in deciding the continuation of treatment with anti-PD-1/L1 inhibitors. Further studies are warranted to confirm the definition of DR, and clarify the mechanism of DR in patients treated with anti-PD-1/L1 inhibitors.

## Data Availability

The datasets analyzed in the present study are not publicly available due to confidential clinical data for individual patients. However, the datasets are available from the corresponding author on reasonable request.

## Abbreviations

Anti-PD-1/L1 inhibitors: Anti-programmed cell death-1/ligand 1 inhibitors
CT: Computed tomography
DR: Dissociated responses
NSCLC: Non-small cell lung cancer
OS: Overall survival
PD: Progressive disease
PR: Partial response
RECIST 1.1: Response Evaluation Criteria in Solid Tumors version 1.1
SD: Stable disease

## References

[1] Brahmer J, Reckamp KL, Baas P, Crinò L, Eberhardt WE, Poddubskaya E (2015). Nivolumab versus Docetaxel in advanced squamous-cell non-small-cell lung Cancer. N Engl J Med.

[2] Borghaei H, Paz-Ares L, Horn L, Spigel DR, Steins M, Ready NE (2015). Nivolumab versus Docetaxel in advanced nonsquamous non-small-cell lung Cancer. N Engl J Med.

[3] Herbst RS, Baas P, Kim DW, Felip E, Pérez-Gracia JL, Han JY (2016). Pembrolizumab versus docetaxel for previously treated, PD-L1-positive, advanced non-small-cell lung cancer (KEYNOTE-010): a randomised controlled trial. Lancet.

[4] Rittmeyer A, Barlesi F, Waterkamp D, Park K, Ciardiello F, von Pawel J (2017). Atezolizumab versus docetaxel in patients with previously treated non-small-cell lung cancer (OAK): a phase 3, open-label, multicentre randomised controlled trial. Lancet.

[5] Schadendorf D, Hodi FS, Robert C, Weber JS, Margolin K, Hamid O (2015). Pooled analysis of long-term survival data from phase II and phase III trials of Ipilimumab in Unresectable or metastatic melanoma. J Clin Oncol.

[6] Katz SI, Hammer M, Bagley SJ, Aggarwal C, Bauml JM, Thompson JC (2018). Radiologic Pseudoprogression during anti-PD-1 therapy for advanced non-small cell lung Cancer. J Thorac Oncol.

[7] Champiat S, Dercle L, Ammari S, Massard C, Hollebecque A, Postel-Vinay S (2017). Hyperprogressive disease is a new pattern of progression in Cancer patients treated by anti-PD-1/PD-L1. Clin Cancer Res.

[8] Tazdait M, Mezquita L, Lahmar J, Ferrara R, Bidault F, Ammari S (2018). Patterns of responses in metastatic NSCLC during PD-1 or PDL-1 inhibitor therapy: comparison of RECIST 1.1, irRECIST and iRECIST criteria. Eur J Cancer.

[9] Dong ZY, Zhai HR, Hou QY, Su J, Liu SY, Yan HH (2017). Mixed responses to systemic therapy revealed potential genetic heterogeneity and poor survival in patients with non-small cell lung Cancer. Oncologist..

[10] Eisenhauer EA, Therasse P, Bogaerts J, Schwartz LH, Sargent D, Ford R (2009). New response evaluation criteria in solid tumours: revised RECIST guideline (version 1.1). Eur J Cancer.

[11] Kanda Y (2013). Investigation of the freely available easy-to-use software 'EZR' for medical statistics. Bone Marrow Transplant.

[12] Kim S, Koh J, Kwon D, Keam B, Go H, Kim YA (2017). Comparative analysis of PD-L1 expression between primary and metastatic pulmonary adenocarcinomas. Eur J Cancer.

[13] Gasteiger G, Ataide M, Kastenmüller W (2016). Lymph node - an organ for T-cell activation and pathogen defense. Immunol Rev.

[14] Chen DS, Mellman I (2017). Elements of cancer immunity and the cancer-immune set point. Nature.

[15] Gettinger SN, Wurtz A, Goldberg SB, Rimm D, Schalper K, Kaech S (2018). Clinical features and Management of Acquired Resistance to PD-1 Axis inhibitors in 26 patients with advanced non-small cell lung Cancer. J Thorac Oncol.

[16] Borcoman E, Kanjanapan Y, Champiat S, Kato S, Servois V, Kurzrock R (2019). Novel patterns of response under immunotherapy. Ann Oncol.

[17] Wang H, Agulnik J, Kasymjanova G, Fiset PO, Camilleri-Broet S, Redpath M (2019). The metastatic site does not influence PD-L1 expression in advanced non-small cell lung carcinoma. Lung Cancer.

[18] Oliver AJ, Lau PKH, Unsworth AS, Loi S, Darcy PK, Kershaw MH (2018). Tissue-dependent tumor microenvironments and their impact on immunotherapy responses. Front Immunol.

[19] Hodi FS, Ballinger M, Lyons B, Soria JC, Nishino M, Tabernero J (2018). Immune-modified response evaluation criteria in solid tumors (imRECIST): refining guidelines to assess the clinical benefit of Cancer immunotherapy. J Clin Oncol.

[20] Wolchok JD, Hoos A, O'Day S, Weber JS, Hamid O, Lebbé C (2009). Guidelines for the evaluation of immune therapy activity in solid tumors: immune-related response criteria. Clin Cancer Res.

[21] Nishino M, Giobbie-Hurder A, Gargano M, Suda M, Ramaiya NH, Hodi FS (2013). Developing a common language for tumor response to immunotherapy: immune-related response criteria using unidimensional measurements. Clin Cancer Res.

[22] Seymour L, Bogaerts J, Perrone A, Ford R, Schwartz LH, Mandrekar S (2017). iRECIST: guidelines for response criteria for use in trials testing immunotherapeutics. Lancet Oncol.

[23] Shukuya T, Mori K, Amann JM, Bertino EM, Otterson GA, Shields PG (2016). Relationship between overall survival and response or progression-free survival in advanced non-small cell lung Cancer patients treated with anti-PD-1/PD-L1 antibodies. J Thorac Oncol.

[24] Ricciuti B, Genova C, Bassanelli M, De Giglio A, Brambilla M, Metro G (2019). Safety and efficacy of Nivolumab in patients with advanced non-small-cell lung Cancer treated beyond progression. Clin Lung Cancer.

